# Validation and Limitations of the PANOMEN-3 Predictive Model for Tumor Recurrence and Progression in Pituitary Tumors

**DOI:** 10.1210/clinem/dgaf252

**Published:** 2025-04-25

**Authors:** Marta Araujo-Castro, Edelmiro Menéndez Torre, Claudia Lozano-Aida, Rogelio García-Centeno, Laura González Fernández, Cindy Idrobo, Estefanía Achote-Rea, Ana Irigaray Echarri, María Dolores Moure Rodríguez-Argulló, Miguel Paja, Fernando Guerrero-Pérez, Justo P Castaño, María Dolores Ollero García, Cristina Novo-Rodríguez, Carmen Tenorio-Jimenéz, Rocío Villar-Taibo, Ignacio Bernabeu, Everardo Díaz-López, María Calatayud, Cristina Alvarez-Escola, Patricia Martín Rojas-Marcos, José María Recio-Córdova, Anna Aulinas, Queralt Asla Roca, María Dolores Aviles, Elena López Mezquita, María Fernández-Argüeso, Inmaculada González Molero, Ignacio Ruz-Caracuel, Iban Aldecoa, Julia García-Arabehety, Elena Martínez-Sáez, Felicia Hanzu, Mónica Marazuela, Manel Puig-Domingo, Betina Biagetti

**Affiliations:** Department of Endocrinology & Nutrition, Hospital Universitario Ramón y Cajal, 28034 Madrid, Spain; Instituto de Investigación Biomédica Ramón y Cajal (IRYCIS), 28034 Madrid, Spain; Department of Endocrinology & Nutrition, Hospital Universitario Central de Asturias, 33011 Asturias, Spain; Instituto de Investigación Sanitaria del Principado de Asturias (ISPA), 33011 Asturias, Spain; Department of Endocrinology & Nutrition, Hospital Universitario Central de Asturias, 33011 Asturias, Spain; Instituto de Investigación Sanitaria del Principado de Asturias (ISPA), 33011 Asturias, Spain; Department of Endocrinology & Nutrition, Hospital Universitario Gregorio Marañón, 28007 Madrid, Spain; Department of Endocrinology & Nutrition, Hospital Universitario Gregorio Marañón, 28007 Madrid, Spain; Department of Endocrinology & Nutrition, Hospital Universitario Ramón y Cajal, 28034 Madrid, Spain; Instituto de Investigación Biomédica Ramón y Cajal (IRYCIS), 28034 Madrid, Spain; Department of Endocrinology & Nutrition, Hospital Universitario Ramón y Cajal, 28034 Madrid, Spain; Instituto de Investigación Biomédica Ramón y Cajal (IRYCIS), 28034 Madrid, Spain; Department of Endocrinology & Nutrition, Hospital Universitario Navarra, 28027 Pamplona, Spain; Department of Endocrinology & Nutrition, Hospital Universitario de Cruces, 48903 Bilbao, Spain; Department of Endocrinology & Nutrition, OSI Bilbao-Basurto, Hospital Universitario de Basurto, 48013 Bilbao, Spain; University of the Basque Country UPV/EHU, 48013 Bilbao, Spain; Department of Endocrinology & Nutrition, Hospital Universitario de Bellvitge, Cataluña L'Hospitalet de Llobregat, 08907 Barcelona Spain; Maimónides Biomedical Research Institute of Córdoba (IMIBIC), University of Cordoba and Reina Sofía University Hospital, 14004 Cordoba, Spain; Department of Endocrinology & Nutrition, Hospital Universitario Navarra, 28027 Pamplona, Spain; Department of Endocrinology & Nutrition, Hospital Universitario Virgen de las Nieves & Biosanitary Research Institute IBS Granada, 18014 Granada, Spain; Department of Endocrinology & Nutrition, Hospital Universitario Virgen de las Nieves & Biosanitary Research Institute IBS Granada, 18014 Granada, Spain; Department of Endocrinology & Nutrition, Hospital Universitario de Santiago de Compostela, 15706 Galicia, Spain; Department of Endocrinology & Nutrition, Hospital Universitario de Santiago de Compostela, 15706 Galicia, Spain; Department of Endocrinology & Nutrition, Hospital Universitario de Santiago de Compostela, 15706 Galicia, Spain; Department of Endocrinology & Nutrition, Hospital Universitario 12 de Octubre, 28041 Madrid, Spain; Department of Endocrinology & Nutrition, Hospital Universitario La Paz, 28046 Madrid, Spain; Department of Endocrinology & Nutrition, Hospital Universitario La Paz, 28046 Madrid, Spain; Department of Endocrinology & Nutrition, Hospital Universitario de Salamanca, 37007 Salamanca, Spain; Department of Endocrinology & Nutrition, Hospital de la Santa Creu i Sant Pau, IR-SANT PAU, EndoERN, CIBERER U747 (ISCIII), 08025 Barcelona, Spain; Department of Endocrinology & Nutrition, Hospital de la Santa Creu i Sant Pau, IR-SANT PAU, EndoERN, CIBERER U747 (ISCIII), 08025 Barcelona, Spain; Department of Endocrinology & Nutrition, Hospital Universitario Clínico San Cecilio, 18007 Granada, Spain; Department of Endocrinology & Nutrition, Hospital Universitario Clínico San Cecilio, 18007 Granada, Spain; Department of Endocrinology & Nutrition, Hospital Universitario de Santiago de Compostela, 15706 Galicia, Spain; Department of Endocrinology & Nutrition, Hospital Regional Universitario de Málaga, 29010 Málaga, Spain; IBIMA Plataforma BIONAND, 29010 Málaga, Spain; Instituto de Investigación Biomédica Ramón y Cajal (IRYCIS), 28034 Madrid, Spain; Department of Pathology, Hospital Universitario Ramón y Cajal & CIBER-ONC, 28034 Madrid, Spain; Department of Pathology Department, Biomedical Diagnostic Center, Hospital Clinic de Barcelona, University of Barcelona, 08036 Barcelona, Spain; Department of Endocrinology & Nutrition, Hospital Universitario Vall de Hebrón, 08035 Barcelona, Spain; CIBERER Group 747, 08035 Barcelona, Spain; Department of Pathology, Hospital Universitario Vall de Hebrón, 08035 Barcelona, Spain; Department of Endocrinology & Nutrition, Hospital Clinic de Barcelona, 08036 Barcelona, Spain; CIBERDEM, IDIBAPS, 08036 Barcelona, Spain; Department of Endocrinology & Nutrition, Hospital Universitario La Princesa, 28006 Madrid, Spain; CIBERER Group 747, 08035 Barcelona, Spain; Department of Endocrinology & Nutrition, Hospital Universitario Germans Trias, 08916 Barcelona, Spain; Department of Endocrinology & Nutrition, Hospital Universitario Vall de Hebrón, 08035 Barcelona, Spain; CIBERER Group 747, 08035 Barcelona, Spain

**Keywords:** pituitary tumors, recurrence, tumoral progression, histopathology classification, risk factors, PANOMEN-3

## Abstract

**Context:**

It has been proposed that the PANOMEN-3 classification may be useful to guide the prognosis and therapy of patients with pituitary tumors (PTs). However, the model has not yet been validated to date in order to assess its usefulness in routine clinical practice.

**Objective:**

The aim of our study was to validate the classification proposed by the PANOMEN-3 group for the prediction of tumor recurrence/progression in PTs.

**Methods:**

Multicenter national case–control study of patients with PTs followed for at least 5 years. Kaplan–Meier curves were used to assess the time to tumor recurrence/progression. Univariate and multivariate Cox regression analyses were used to estimate the hazard ratio (HR) and prognostic capacity of the classification proposed by the PANOMEN-3 group.

**Results:**

A total of 1143 patients were included. Pituitary surgery was performed in 814 patients and the remaining 329 patients were followed with active surveillance or medical treatment. After a median follow-up of 8.8 years (5-29.8), 253 patients experienced tumor recurrence or biochemical/radiological progression and were classified as cases. The other 890 patients were classified as controls. The mean follow-up from PT diagnosis to recurrence was 7.2 ± 5.4 years. The diagnostic accuracy of the PANOMEN-3 model to predict recurrence/progression was 75.6% (95% CI 0.716-0.796). Residual tumor (HR 2.20, *P* < .001), a hereditary syndrome (HR 5.15, *P* = .026), and active secretory status (HR 1.80, *P* = .021) were the most important variables in this model. Recurrence/progression rate increased with increasing PANOMEN-3 grade (2.5% in grade 0; 10.3% in grade 1, 33.7% in grade 2, and 33.3% in grade 3; *P* < .001).

**Conclusion:**

The predictive model proposed by the PANOMEN-3 group may be useful to guide the prognosis and therapy of PTs in the Spanish population since it offers a good accuracy to predict tumoral/biochemical recurrence and/or progression in operated and nonoperated patients.

Pituitary tumors (PTs) are common indolent neoplasms, with a prevalence of 10% in autopsy series (1) and of 4% to 20% in radiologic studies (2). Although these tumors usually present an indolent evolution, there is a marked heterogeneity in their clinical presentation and biological behavior depending on several factors such as the specific PT subtype based on hormonal or histopathological classifications or tumor extension/invasiveness, among others (1). Furthermore, although rare, cases of metastatic development or aggressive progression have been described (3-5), and this is the main reason of why the latest World Health Organization (WHO) classification advocates the use of the term pituitary neuroendocrine tumor instead of pituitary adenoma (6). However, the real situation today is that more than half of all PTs that cause clinically significant health problems do not require surgical resection. Therefore, these patients lack histological data that would facilitate pathological classification of tumor subtype and aid in recurrence prediction. For this reason, in 2024 a group of experts from the Pituitary Society (PANOMEN-3 group) proposed the development of a score that integrates the clinical, genetic, biochemical, radiologic, histopathologic, and molecular data of all tumors arising from anterior pituitary cell lineages to guide their management and provide information about their long-term prognosis (7). They claimed that this new classification may be useful to guide the prognosis and therapy of patients with PTs. However, this model was developed based on expert opinion and has not been validated to date in order to assess its usefulness in routine clinical practice.

In this context, the aim of our study was to validate the classification proposed by the PANOMEN-3 group, with the ultimate goal to determine whether this combined clinic–pathologic classification could be useful to identify those patients with PTs with a higher probability of tumor or biochemical progression or tumor recurrence during follow-up.

## Material and Methods

### Study Design

This was a national Spanish multicenter observational cohort study in which 17 Spanish tertiary hospitals participated. Patients with PTs were consecutively enrolled from the endocrinology departments of the participating centers. Patients were followed for a minimum of 5 years or until tumor recurrence/progression was detected, whichever occurred first. Patients were identified from the local register/database of the pituitary/neuroendocrinology consultation of each participant center, selecting a period between of consultations between 2015 and 2019, and ensuring that all patients who accomplish the inclusion criteria were consecutively included. We followed the STROBE statement for the design of our study (8).

Inclusion criteria for the study (PIT-VALIDATION study) were to have a PT that met at least 1 of the following criteria (Table 1): (1) biochemical diagnosis of prolactinoma (9); (2) biochemical diagnosis of Cushing disease (10), (3) biochemical diagnosis of acromegaly (11), (4) biochemical diagnosis of thyrotropin (TSH) secreting PT (12), or (5) radiological and/or pathological diagnosis of PT with hormonal study in which functionality was ruled out. We excluded those cases with a follow-up shorter than 5 years, with incomplete clinical, radiological, histopathological, or follow-up information, and with histopathological diagnosis other than pituitary adenoma.

**Table 1.** Inclusion and exclusion criteria to enter in the PIT-VALIDATION study

Inclusion criteria	Exclusion criteria
Prolactinoma diagnosis Cushing disease diagnosis Acromegaly diagnosis TSH secreting PTs diagnosis Radiological and/or pathologic diagnosis of PT with hormonal study in which functionality was ruled out	Follow-up time shorter than 5 years Incomplete clinical, radiologic, pathologic, or follow-up information Histopathologic diagnosis other than pituitary adenoma/pituitary neuroendocrine tumor

Abbreviations: PT, pituitary tumor; PRL, prolactin; TSH, thyrotropin.

The study was endorsed by the Spanish Society of Endocrinology and Nutrition (SEEN). The local ethics committee of the Ramón y Cajal University Hospital reviewed and approved the study (approval date: May 28, 2024, code: ACTA 467). The study was conducted according to the mandates of the Declaration of Helsinki and good clinical practices. Informed consent was obtained for all the patients with active follow-up. For patients included retrospectively, informed consent was waived if they were not on active follow-up or could not be contacted, in accordance with ethical guidelines and local regulations.

In summary, the main outcome of our study was to perform an independent external validation of the prognostic classification proposed by Ho et al (PANOMEM-3 classification) with the aim of assessing the prognostic capacity of this classification to predict recurrence and biochemical/tumoral progression in PTs (7).

### Definitions

The main analysis (disease-free after at least 5 years of follow-up) compared patients with recurrence/progression (cases) to those without evidence of disease/progression (controls) during the 5-year follow-up period. Follow-up time was considered since the diagnosis of the PT until the last available visit (in controls) or until recurrence/progression (in cases).

**Figure 1.**
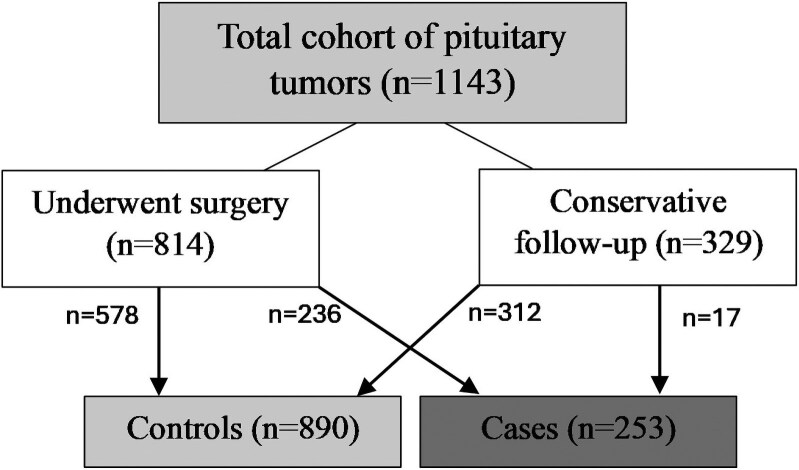
Study population.

We classified as controls those patients in complete remission after pituitary surgery with no evidence of active disease (biochemical and radiological) or with a residual tumor with no significant growth during at least 5 years of follow-up. For nonoperated patients, we classified them as controls if there was no evidence of hormonal excess and tumor progression while under medical treatment in functioning PTs and as tumor stability in nonfunctioning pituitary tumors (NFPTs). We classified as cases those nonoperated patients with biochemical or tumor progression; and in the operated patient's group, those who presented an increasing tumor remnant (functioning and nonfunctioning) and those with biochemical recurrence (functioning). Based on this definition, 253 patients were classified as cases and 890 as controls (Fig. 1).

Tumor size was considered stable if tumor growth or the increase in tumor size of the tumor was <20% or <2 mm from basal tumor size and the growth was not associated with worsening of symptoms or appearance of new symptoms. Significant tumor growth was considered if the tumor size increased by ≥20% or ≥2 mm in a 2-year period and/or new symptoms of mass effect occurred (13). Biochemical progression was defined as a ≥30% increase in urinary free cortisol (Cushing disease), insulin-like growth factor-1 (acromegaly) or prolactin (prolactinoma) compared with previous values on treatment that required an increase in the dose and/or number of drugs for an adequate biochemical control (14-17).

### Variables

A specific registry (PIT-SCORE database, available in REDCAP software) was set up to collect information on clinical characteristics including gender, age, and hereditary syndromes, radiologic data, treatment (surgical and/or medical therapies and response), and histopathologic information. Radiological evaluation was performed using magnetic resonance imaging and data on maximum tumor diameter, cavernous sinus invasion (Knosp grade), and hypointensity or iso/hyperintensity in the T2 sequence were recorded. Cavernous sinus invasion was graded using the Knosp–Steiner classification based on coronal T1-weighted contrasted imaging (18). Based on maximum tumor diameter, tumors were categorized as microadenoma (<1 cm), macroadenoma (1-4 cm), and giant PTs (≥4 cm). Hormonal and radiological information was collected at the time of the PT diagnosis and at the last follow-up visit.

Regarding histopathologic information, the following features were included in the description of the pathologic specimen: immunostaining for human growth hormone, prolactin, TSH, adrenocorticotropin (ACTH), follicle-stimulating hormone, luteinizing hormone, and Ki-67. Information on the expression of the different transcription factors (PIT1, TPIT, SF1, GATA3, and Erα) was available in 207 cases (19), and these cases were classified according to the 2022 WHO classification (20). Based on this WHO classification and following the proposed by the PANOMEN-3 group, we classified the immature PIT1 lineage, Crooke cell, null cell, silent corticotroph PT, sparsely granulated somatotroph, and acidophilic stem cell adenoma as high-risk histological subtypes (19, 21). Increased proliferation was defined by a Ki-67 >10% or a mitotic index is at least 2 mitoses per 10 high power fields

For the validation of the PANOMEN-3 prediction model (7), we grouped the variables according to its 9 items: (1) phenotype (0 = NFPTs and prolactinomas, 1 = acromegaly and TSH secreting PTs, and 2 = Cushing disease); (2) secretory status (0 = normal or biochemically controlled, 1 = elevated), (3) hypopituitarism (0 = absent, 1 = partial without antidiuretic hormone (ADH) deficiency and 2 = hypopituitarism with ADH deficit), (4) size (0 = micro, 1 = macro, 2 = giant PT), (5) mass effect (0 = absent, 1 = visual defects or cranial nerve palsy or cerebrospinal fluid leakage), (6) invasion (0 = absent, 1 = Knosp 3 or 4), (7) residual tumor (0 = absent, 1 = present), (8) histopathology (1 = high-risk subtype, 1 = increased proliferation), and (9) genetic syndrome (0 = absent, 1 = present).

Following the PANOMEN-3 score, we calculated the corrected score as the cumulative raw score of these 9 items divided by the number of risk factors rounded to a single decimal. Then, based on the corrected score patients we assigned to 4 different grades: grade 0 if corrected score is equal to 0; grade 1 if it is more than 0 and less than 0.3; grade 2 if it is between 0.3 and 0.6; and grade 3 if it is more than 0.6.

### Statistical Analysis

Statistical analysis was performed using STATA.15. In the descriptive analysis, categorical variables were expressed as percentages and absolute values of the variable; quantitative variables were expressed as mean ± SD or as median and range depending on whether the normality assumption was met. To compare differences in baseline continuous variables between 2 subgroups we used the Student t test, and chi square (χ^2^) test to compare baseline categorical data.

To describe the time of tumor recurrence/progression, the cumulative incidence was estimated using the Kaplan–Meier method. The univariate Cox proportional hazards model was used to estimate crude hazard ratios (HRs), and the multivariate Cox proportional hazards model was used to estimate multivariable-adjusted HRs with 95% CIs, as well as for evaluating possible predictors of recurrence. To assess the prognostic capacity of the classification proposed by Ho to predict recurrence in PTs, survival analysis was used. The concordance index (C-index), calibration curve, and receiver operating characteristic were used to determine the predictive accuracy of the nomogram. Decision curve analysis was performed to evaluate the clinical efficacy of the nomogram.

Based on sample size estimation, the minimum number of patients to be included to detect a 10% incidence of tumor recurrence/progression with a power of 0.9 and an error of 0.05 was 540 patients (54 in the progression/recurrence group and 486 in the nonprogression/nonrecurrence group).

## Results

### Study Population

**Figure 2.**
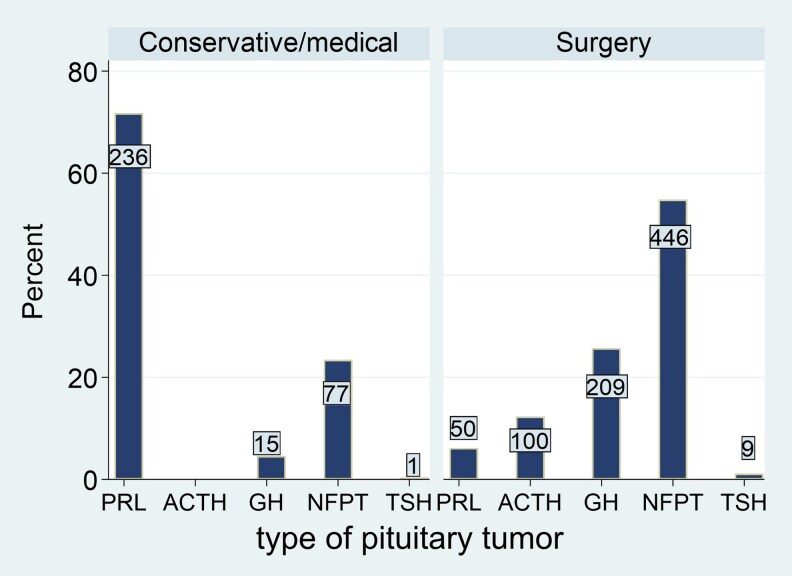
Proportion of the different pituitary tumors in the surgical and conservative group.

A total of 1143 patients with PT were included in the study, 523 were males and 620 females. The mean age at diagnosis was 49.4 ± 16.4 years. Nine patients were registered as having a hereditary syndrome associated with PT (7 patients with MEN1, 1 with MEN4, and another with familial acromegaly). Most of the patients underwent pituitary surgery (n = 814, 71.2%) and the remaining 329 patients (28.8%) were actively followed without specific treatment (n = 71) or were medically treated (n = 258). Of these 258 patients treated with first-line medical therapy, 14 had acromegaly, 11 had NFPTs (dopamine agonist treatment), 232 had prolactinomas, and 1 had a TSH-secreting PT. A total of 162 patients received radiation therapy during follow-up. Surgery was more frequently performed in older patients, those with pituitary hormonal deficiency at diagnosis, headache, visual involvement, macroadenomas, and higher Knosp grade (Table 2). In addition, patients who underwent surgery were more likely to receive postoperative radiotherapy than those who did not. The baseline characteristics of the patients, including those who underwent surgery and those who were managed conservatively, are described in Table 2. Figure 2 shows the distribution of PTs included in the study. When we analyzed the proportion of high-risk histological subtypes in these subtypes of PT, the proportion of these high-risk histological subtypes was higher in NFPTs (25.3%), growth hormone–secreting PTs (18.9%), and prolactinomas (16.7%) than in patients with Cushing disease (6.2%) (*P* < .001).

**Table 2.** Baseline characteristics of the cohort study

Variable	General cohort (n = 1143)	Underwent surgery (n = 814)	Conservative management (n = 329)	*P* value
Age (years)	49.4 ± 16.4	52.2 ± 14.8	42.6 ± 18.1	<.001
Female sex	54.2% (n = 620)	51.7% (n = 421)	60.5% (n = 199)	.007
Hypopituitarism	37.2% (n = 425)	37.8% (n = 308)	35.6% (n = 117)	.471
ACTH deficit	15.7% (n = 179)	19.0% (n = 155)	7.3% (n = 24)	<.001
TSH deficit	18.2% (n = 208)	22.1% (n = 180)	8.5% (n = 28)	<.001
LH/FSH deficit	31.3% (n = 358)	30.7% (n = 250)	32.8% (n = 108)	.485
GH deficit	11.3% (n = 129)	13.0% (n = 106)	7.0% (n = 23)	.004
Visual defects	35.4% (n = 404)	45.1% (n = 367)	11.3% (n = 37)	<.001
Visual fields	29.9% (n = 342)	38.6% (n = 314)	8.5% (n = 28)	<.001
Visual acuity loss	10.8% (n = 123)	13.6% (n = 111)	3.7% (n = 12)	<.001
Cranial nerves	3.9% (n = 45)	4.8% (n = 39)	1.8% (n = 6)	.020
Headache	29.3% (n = 335)	33.8% (n = 275)	18.2% (n = 60)	<.001
Macroadenoma	76.7% (n = 877)	85.3% (n = 694)	55.6% (n = 183)	<.001
Tumor size (mm)	20.2 ± 12.48	22.7 ± 12.20	14.1 ± 11.00	<.001
Right Knosp ≥2 (n = 1073)	31.3% (n = 336/1073)	37.0% (n = 279/754)	17.9% (n = 57/319)	<.001
Left Knosp ≥2 (n = 934)	30.4% (n = 328/1078)	38.1% (n = 289/758)	12.2% (n = 39/320)	<.001
T2 hypointensity (n = 877)	37.6% (n = 330/877)	38.2% (n = 237/621)	36.3% (n = 93/256)	.610
Radiotherapy	14.4% (n = 162)	19.5% (n = 159)	1.0% (n = 3)	<.001
Follow-up time (years)	9.8 ± 5.37	9.8 ±5.15	10.0 ± 5.87	.572

Abbreviations: ACTH, adrenocorticotropin; FSH, follicle-stimulating hormone; GH, growth hormone; LH, luteinizing hormone; TSH, thyrotropin.

### Tumoral Recurrence and Progression

After a median follow-up of 8.8 (range 5-29.8) years, there were 253 (22.1%) patients who experienced tumor recurrence or biochemical/radiologic progression and were thus classified as cases (236 in the surgical group and 17 in the conservative/medical treatment group). Of the 253 cases, 41 had tumor recurrence after surgical cure, 166 had significant tumor growth, and 46 had functioning PTs with significant biochemical progression. Four out of the 166 cases with significant tumor progression developed pituitary metastases after a median follow-up of 11.0 years (range -20) from PT diagnosis. Regarding the PT subtypes of these 4 metastatic tumors, 2 had prolactinoma, 1 had NFPT, and another had ACTH-secreting PT. All of them underwent surgery but residual tumor after surgery was present, and they also received radiotherapy and temozolomide during follow-up. In relation to histological data of these 4 patients, 2 were lactotroph tumors (prolactinoma cases) and the other 2 had corticotroph tumors (1 of them a silent corticotroph tumor). The Ki-67 index in these patients was 2.7%, 8%, 20%, and 14% respectively.

Overall, the recurrence rate was 27.2% in NFPTs and 17.9% in functioning PTs (9.8% in prolactinomas, 17.0% in acromegaly/TSH-secreting PT, and 41% in Cushing, *P* < .001). The median follow-up from PT diagnosis until recurrence was 7.2 ± 5.4 years. The mean follow-up time was longer in cases than in controls (11.5 ± 5.71 vs 9.3 ± 5.2 years, *P* < .001).

**Figure 3.**
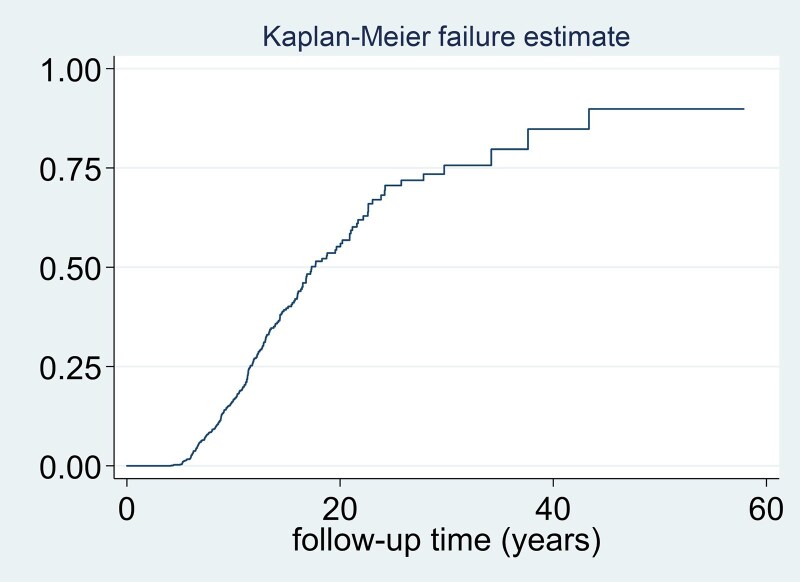
Kaplan–Meier curve of recurrence and tumoral/biochemical progression of pituitary tumors during follow-up. The graph shows the cumulative tumor recurrence or progression rate over 50 years of follow-up, with the recurrence/progression rate (0-1.0) on the y-axis and the follow-up time in years in x-axis. The median recurrence-free survival time was 17.3 years (95% CI 16.4-20.2), indicating that 50% of patients experienced a recurrence within this time period.

**Figure 4.**
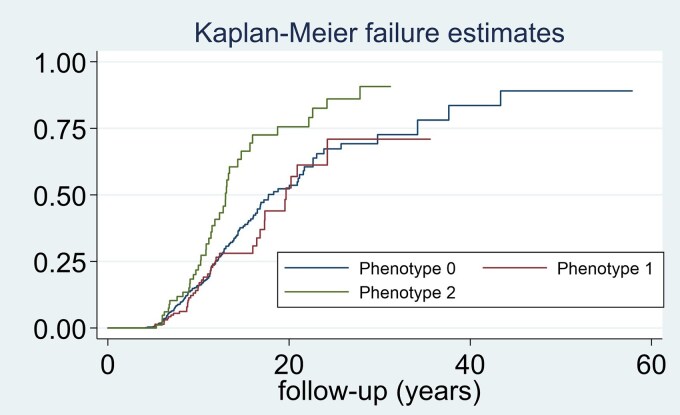
Kaplan–Meier curve of recurrence and tumoral/biochemical progression of pituitary tumors during follow-up based on PANOMEN-3 phenotype. The Kaplan–Meier curves illustrate the failure rates over a follow-up period of up to 50 years for 3 different phenotype groups: Phenotype 0 (nonfunctioning and prolactinoma), Phenotype 1 (acromegaly and thyrotropin-secreting pituitary adenoma), and Phenotype 2 (Cushing disease). The y-axis represents the cumulative failure probability, while the x-axis shows the duration of follow-up in years. Phenotype 2 exhibits the fastest rate of failure, as indicated by its steep curve.

Figure 3 shows the Kaplan–Meier curve of progression/recurrence during follow-up in our sample. In the early years of follow-up, there was a relatively low rate of recurrence, as evidenced by the flat curve, and higher incidence was observed in the 18- to 24-year period, as reflected by the steepest slope of the Kaplan–Meier curve and the HR of 0.192 cases/patient-year (192 cases/100 patient-years) during this interval (Table 3 and Fig. 3). When we compared recurrence rate in the different PT according to secretory phenotype, the higher rate of recurrence was observed in patients with Cushing disease and the lower rate in patients with prolactinomas (Table 4 and Fig. 4). This risk was higher in the surgical group than in the conservative management group (log rank test for trend *P* < .001). At the last follow-up, 39 patients had died; 6 of 251 (2.4%) in the case group and 33 of 866 (3.8%) in the control group (*P* = .280). Only 1 out of the 39 deaths were related to pituitary disease (Cushing syndrome in 1 patient). The median recurrence-free survival was 16.5 years (95% CI 14.9-18.7), indicating that 50% of patients experienced a recurrence within this period of time.

**Table 3.** Incidence of recurrence during follow-up in the global cohort

Interval (years)	Total	Cumulative failure	Hazard	95% CI
0-6	1143	0.004	0.001	0.000-0.012
6-12	1060	0.133	0.023	0.019-0.028
12-18	372	0.293	0.034	0.025-0.043
18-24	159	0.810	0.192	0.160-0.224
24-30	26	0.857	0.048	0.000-0.140
54-60	1	0.857	0.046	NC

Abbreviation: NC, not calculable.

**Table 4.** Risk factors for recurrence and tumoral/biochemical progression

Variable	Hazard ratio	95% CI	*P* value
Age at diagnosis (years)	1.23 per each 10 years	1.13-1.34	<.001
Male sex	1.40	1.09-1.79	.009
PANOMEN-3 phenotype 2 vs 0	1.80	1.28-2.54	.002
PANOMEN-3 phenotype 1 vs 0	0.86	0.62-1.21	.391
Prolactinoma	0.23	0.16-0.35	<.001
Secretory status	1.74	1.25-2.42	.002
Partial deficit	1.15	0.87-1.52	.318
Panhypopituitarism	1.23	1.02-1.49	.042
Macroadenoma vs micro	1.96	1.38-2.79	<.001
Giant PT vs micro	1.53	1.18-1.99	.021
Tumor size (mm)	1.16 per each 10 mm	1.08-1.26	<.001
Mass effect symptoms	1.53	1.19-1.96	.001
Knosp ≥2	2.13	1.63-2.79	<.001
Residual tumor	2.23	1.65-3.03	<.001
High-risk histopathology	0.85	0.59-1.22	.372
Ki67 > 3% or positive p53	1.20	0.82-1.78	.348
Genetic syndrome	1.34	0.43-4.18	.635

Abbreviation: PT, pituitary tumor.

### Validation of the PANOMEN-3 Predictive Model

In the univariate analysis, the variables associated with a higher rate of recurrence/progression were an older age at diagnosis, male sex, having an ACTH-secreting PT, hormonally active disease, having a macroadenoma, tumor size (mm), Knosp >2, and postoperative tumor remnant. On the other hand, the diagnosis of prolactinoma was associated with a lower risk of recurrence (Table 4). The association of recurrence with age disappeared when we adjusted for the type of PT (adjusted HR 1.01, 95% CI 1.00-1.02, *P* = .069). In this regard, when we analyzed the influence of age in the different types of PT, the only subtype in which an older age was associated with a higher risk of recurrence/progression was prolactinomas (adjusted HR 1.42 per each 10-year increase, 95% CI 1.08-1.88, *P* = .015). The association between older age and prolactinoma recurrence persisted after adjustment for sex (adjusted HR 1.40 per each 10-year increase, 95% CI 1.03-1.91, *P* = .031).

When we tested the Ho et al model in our population (PANOMEN-3 classification), the accuracy of this model to predict recurrence/progression was 75.6% (area under the receiver operating characteristic curve 0.756, 95% CI 0.716-0.796). The most important variable of the model was the presence of a hereditary syndrome (HR 5.15), followed by postsurgical residual tumor (HR 2.20), and active vs nonactive secretory status (HR 1.80) (Table 5). It should be noted that the risk of recurrence increased as larger postsurgical tumor rest was detected (HR 1.19 per each centimeter increase, 95% CI 1.02-1.38). For grade 3, the positive predictive value was 33.3% and negative predictive value was 79.2%; and for grade 2, the positive predictive value was 32.8% and the negative predictive value was 90.9%.

**Table 5.** Predictive value of the PANOMEN-3 classification in a PT Spanish cohort

Variable	Hazard ratio	95% CI	*P* value
PANOMEN-3 score			
Acromegaly/TSH-PTs	0.53	0.33-0.86	.011
Cushing disease	1.30	0.75-2.24	.350
Secretory status	1.80	1.10-2.97	.021
Hypopituitarism			
No ADH deficit	1.07	0.76-1.51	.700
With ADH deficit	1.02	0.64-1.64	.925
Tumor size			
Macroadenoma	1.58	0.79-3.16	.200
Giant pituitary tumor	1.57	0.67-3.68	.298
Mass effect	0.93	0.65-1.32	.674
Invasion (Knosp >2)	1.03	0.73-1.44	.885
Residual tumor	2.20	1.47-3.30	<.001
High-risk histology vs others	0.77	0.53-1.13	.178
Genetic syndrome	5.15	1.22-21.73	.026

Abbreviations: ADH, antidiuretic hormone; PT, pituitary tumor; TSH, thyrotropin.

**Figure 5.**
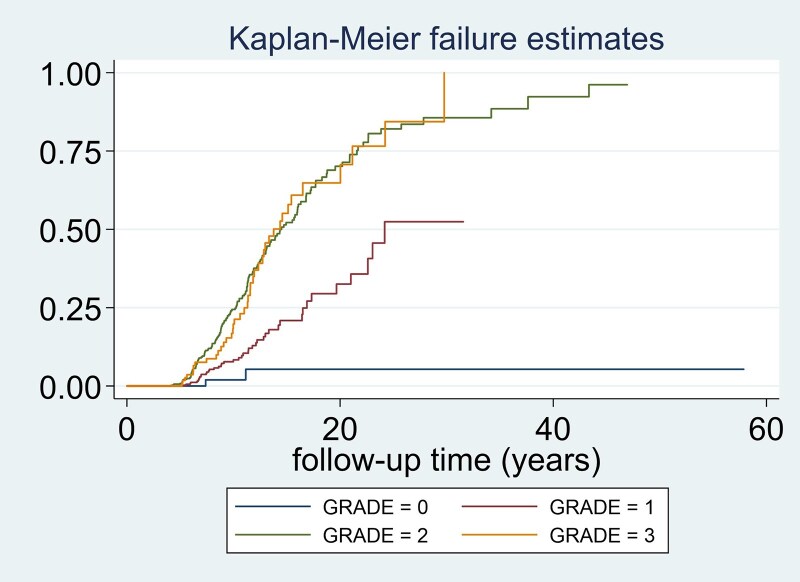
Kaplan–Meier recurrence estimates for tumor recurrence stratified by PANOMEN-3 risk grades. Kaplan–Meier survival curves illustrating the probability of survival (being free of recurrence) over time, stratified by the risk grades assigned according to the PANOMEN-3 classification. Risk grades range from 0 (lowest risk) to 3 (highest risk). Patients with higher grades (eg, grade 3) have shorter survival, reflecting a higher risk of tumor recurrence earlier in the follow-up period. The rate of recurrence/progression increased with increasing PANOMEN3 grade (2.5% in grade 0; 10.3% in grade 1, 32.7% in grade 2 and 33.3% in grade 3) although the discriminatory capacity between grade 2 and grade 3 is limited and could potentially justify merging them into a single grade.

The risk of recurrence based on the total corrected score and grade obtained in this classification is described in Table 6. There was a significantly positive tendency to increase the incidence of recurrence as the PANOMEN-3 grade increased (Mantel–Haenszel test for linear trend: χ^2^ = 78.78, *P* < .001). The differences in survival were evident when comparing the 4 grades (PANOMEN 0, 1, 2, and 3), especially between grade 0 and 1 compared with 2 and 3 (Fig. 5). A significant difference in survival was observed when grade 0 and 1 were compared (logrank test; χ^2^ = 5.80, *P* = .016) and between grade 1 and 2 (logrank test; χ^2^ = 48.22, *P* < .001). However, when we compared survival curves of grades 2 and 3, no statistically significant differences were observed (logrank test; χ^2^ = 0.04, *P* = .847).

**Table 6.** Risk of recurrence/tumor progression based on the grade obtained in the PANOMEN-3 classification

Total Score	Proportion of patients with this score	Proportion of cases (recurrence or progression)
0	6.9% (n = 79)	2.5% (n = 2/79)
1	38.2% (n = 436)	10.3% (n = 45/436)
2	44.2% (n = 505)	32.7% (n = 165/505)
3	10.8% (n = 123)	33.3% (n = 41/123)

Following the PANOMEN-3 score, the table shows the cumulative raw score divided by the number of risk factors rounded to a single decimal = corrected score). The grade was assigned according to corrected score: grade 0 = 0; grade 1 = more than 0 and less than 0.3; grade 2 = between 0.3 and 0.6; grade 3 = more than 0.6.

## Discussion

PTs are common findings in both clinical practice and incidental radiological studies, yet their biological behavior remains highly heterogeneous. This variability, ranging from indolent tumors to aggressive neoplasms with metastatic potential, highlights the need for effective prognostic tools to guide treatment and predict outcome. This is especially critical when histopathological evaluation is unavailable. The PANOMEN-3 classification model, proposed in 2024 (7), represents a significant step forward in integrating multiple dimensions of tumor biology characteristics, including clinical, radiologic, genetic, and histopathologic data. However, its clinical utility remained untested until this study. Here, we present the first external validation of the PANOMEN-3 classification in a large, multicenter cohort of Spanish patients, including both operated and nonoperated patients, providing important insights into its prognostic accuracy and real-world applicability. We demonstrated that the PANOMEN-3 model has a fairly good diagnostic accuracy of 76% for this purpose. As the corrected score/grade of the PANOMEN 3 classification increased, so did the risk of progression/recurrence also increase, from a risk of 2.5% in grade 0% to 33% in grade 3, although the discriminatory capacity between grade 2 and 3 is minimal, and could even be reclassified in a single grade, thus eliminating grade 3, which does not add additional predictive power. This level of diagnostic accuracy is clinically meaningful and supports the ability of this model to effectively stratify patients into risk categories. The strongest predictors of recurrence included hereditary syndromes (HR 5.15), residual tumor (HR 2.20), and active secretory status (HR 1.80), but even with a substantially high number of patients in the different hypersecreting PT cases included in the present study (Cushing n = 100 and acromegaly n = 224), it does not discriminate acromegaly and Cushing cases for risk of recurrence. These results validate—with the aforementioned limitations of the hyperfunctioning tumors—the expert consensus used to develop the model and underscore its relevance to routine clinical practice.

In our study, 22% of the patients experienced recurrence or progression after a median follow-up of 8.1 years. The incidence of recurrence was not uniform throughout the follow-up period. The slope of the Kaplan–Meier curve, indicating the highest incidence, occurred during the 18- to 24-year period. In agreement with our results, the metanalysis by Fernández-Balsells (22) reported that after a median follow-up of 3.9 years (range 1-15) the incidence of significant tumor growth in NFPTs was 12.53 (7.86-17.20) cases per year. One of the largest series included in this metanalysis was the Kim et al series (23), in which a total of 197 patients with NFPTs were followed for a median of 37 months. In this study, up to 44% (n = 87) of the cases experienced tumor growth but only 2.5% developed visual symptoms and 3% pituitary apoplexy. In addition, only 14% in the macroadenoma group and 5% in the microadenoma group had tumor size increase ≥20% after 1 year of follow-up. Imran et al (24) reported also similar findings to our series with an increase in size of ≥2 mm in 20% of the 99 patients with NFPTs at 3 years. Although there are fewer data on recurrence in functioning PTs, we found recurrence/progression in 18% of the global cohort of functioning PTs. A recent study including 122 patients with Cushing disease found a recurrence rate of 38.1% after a median follow-up of 103 months (25). In acromegaly the recurrence rate varies from 1% to 20% to 25% (26, 27), and in patients with prolactinoma, up to 30% of the patients recurred after withdrawal of dopamine agonist (28). However, it is important to highlight that the reported rates of recurrence and progression among the different studies were highly variable because different definitions of recurrence/progression were used and the populations were also quite variable (ie, proportion of macro/micro, functioning/nonfunctioning, or histopathology type).

It is worth mentioning that until the development of the PANOMEN-3 score, there was no comprehensive classification system to guide the prediction of recurrence for patients with pituitary adenomas who had not undergone surgery (7). While the Trouillas et al classification (29) has been until now the most commonly used for prognostic assessment, it could only be applied to surgical patients with available histopathological material. This later classifies PTs in 5 grades based on their invasion and proliferation characteristics (grade 1a: noninvasive tumor, grade 1b: noninvasive and proliferative tumor, grade 2a: invasive tumor, grade 2b: invasive and proliferative tumor, grade 3: metastatic tumor), stratifying for each of the hormone-secreting categories. In addition, it should be noted that the main outcome of the Trouillas classification is to predict disease-free status (vs evidence of disease, including both persistence and recurrence); thus, the proportion of cases originally described was as high as 47.6%. Nevertheless, they also performed a secondary analysis focused on recurrence/progression-free status at 8 years and reported a recurrence/progression rate of 30.7%. They found that age at initial surgery (*P* = .048), tumor type (*P* < .001), and tumor grade (*P* < .001) were associated with patient progression/recurrence status at 8 years. The Trouillas score has been validated by several groups, confirming its clinical value also for predicting recurrence (30, 31). In contrast to the variables included in the Trouillas classification, the results of our study revealed that the most important prognostic factors in the PANOMEN-3 model were the presence of residual tumor (HR 2.20), a hereditary syndrome (HR 5.15), and active secretory status (HR 1.80), rather than tumor histopathology. A hallmark of our study is that the model was tested in surgically treated and conservatively managed patients, as allowed by PANOMEN-3. This highlights its versatility and supports its use across different management approaches. Notably, the risk of recurrence was higher in the surgical group than in the conservative group, likely reflecting the more aggressive nature of tumors requiring surgical intervention. Nonetheless, PANOMEN-3 did not include information about proliferation, such as the Trouillas score, and this may be a limitation since the predictive value of this variable was high in the Trouillas classification (29). However, in our cohort, proliferation defined by a Ki-67 index >3% or positive p53 was not a significant predictive factor of recurrence.

Regarding tumor phenotype and risk of recurrence, the PANOMEN-3 classification assigned a risk score of 2 in Cushing disease, 1 in acromegaly and TSH-secreting PTs, and 0 for prolactinoma and NFPTs. However, it should be kept in mind that this score assignment was based on the higher mortality risk in patients with Cushing disease, followed by acromegaly and TSH-secreting PTs (7), rather than the recurrence risk itself for each disease. Nevertheless, our findings support this stratification. In particular, our results validate the group of prolactinomas as a low-risk PT since the risk of recurrence was 3 times lower in patients with prolactin-secreting PT (all of them macroadenomas, since having a microprolactinoma was an exclusion criterion for our PIT-VALIDATION study) than in the other subtypes. On the other hand, the highest-risk phenotype was Cushing, since the rate of recurrence/progression was 40% in our series compared with 10% to 17% in the other subtypes of PTs. This observation is in agreement with that reported by other authors who reported recurrence in about 20% to 30% of patients with Cushing disease initially cured (32). The proportion in our series was higher since in the group we also included of cases noncured patients with Cushing disease who experienced a significant biochemical and/or tumoral progression during follow-up.

Residual tumor is a significant risk factor for recurrence, as highlighted by the PANOMEN-3 group (7), which identified visible residual tumor on magnetic resonance imaging after pituitary surgery as a reliable predictor of progression. Along these lines, the prospective study by Raverot et al (33), based on the clinicopathological classification by Trouillas et al (29), found that grade 2b tumors had a 3.7-fold increased risk of recurrence or progression compared with grade 1a tumors. Overall, the recurrence rates are around 10% to 20% at 5 years and 30% at 10 years after complete resection, while residual tumors after surgery carry a recurrence risk of 25% to 40% at 5 years and more than 50% at 10 years (34). A meta-analysis by Chen et al (35) reported recurrence rates of 12% for complete resection, and 46% for patients with residual tumor. These findings further support the need for close imaging and hormonal surveillance in patients with residual tumor.

A hereditary syndrome increased the risk of recurrence/progression by a factor of 5. Although the proportion of hereditary PTs is low (about 5%) (36), the identification of these cases is crucial not only because of their higher risk of developing tumors and manifestations in other extrapituitary sites, but also for the more aggressive or treatment-resistant PTs.

Active secretory status is another important point, since one of the main goals in the treatment of functioning PTs is to achieve adequate hormonal control in order to reduce morbidity and mortality (37). Regarding tumor size, some authors reported a higher risk of recurrence in macroadenomas than in microadenomas (34). Moreover, a larger size is associated with a higher likelihood of morbidity, including optic nerve compression, cranial nerve palsies, and headaches, but also of mortality, especially in macrocorticotropinomas. Larger tumor size is usually associated with a higher Knosp grade. This association is important since the Knosp classification provides a good guide for estimating the possibility of surgical cure (38), and, thus, the likelihood of residual tumor rest, which, as mentioned, is one of the most important predictors of recurrence. For instance, in our local series (from the Ramón y Cajal Hospital that is a Pituitary Tumor Center of Excellence) of 228 PTs, surgical cure was lower in invasive PTs than in noninvasive PTs (28.8% vs 83.1%, *P* < .001), and the risk of major complications was higher (13.8% vs 3.4%, *P* = .003) (38).

Regarding age, we found that older age at diagnosis was associated with a higher risk of recurrence in prolactinoma. In this regard, it has been previously described that the influence of age on PT behavior depends in part on the PT subtype. For instance, for acromegaly and NFPT, younger patients tend to have a more active disease, whereas in patients with Cushing disease, morbidity and mortality tend to be higher (39). Although prolactin-secreting tumors generally have a low risk of recurrence, younger men with prolactinomas are often described as having more aggressive disease (40). Interestingly, our study found a higher risk of recurrence in older patients. While this may be partly related to a higher proportion of men in the older group, other factors are likely to contribute, as the association between age and recurrence persisted after adjustment for sex.

The validation of the PANOMEN-3 classification represents a significant advance in the management of PTs, providing clinicians with a reliable tool for risk stratification and personalized treatment planning. Our results demonstrate that this classification system effectively identifies patients at different risk levels, allowing for more targeted and active therapeutic interventions. In particular, patients classified as grades 2 and 3 warrant closer surveillance, with monitoring intervals potentially as frequent as every 3 to 6 months, and may benefit from early consideration of adjuvant therapy. In contrast, grade 0 and grade 1 patients can be safely monitored less frequently, such as annually, optimizing health care resources while maintaining quality of care.

Furthermore, our validation highlights the critical prognostic significance of residual tumor and active secretory status, as previously accepted in usual clinical practice. This emphasizes 2 key clinical priorities: first, the importance of pursuing maximal safe tumor resection when surgery is indicated, and, second, the need for close biochemical monitoring and prompt adjustment of medical therapy to achieve hormonal control.

We are aware that our study has several limitations. One is related to its retrospective design, which must be considered when interpreting the results. In addition, the operations were performed by different surgeons with varying skills in skull base surgery, this could affect the probability of surgical cure. Another limitation is related to genetic studies, since although many patients were classified as nongenetically syndromic, most of the patients did not undergo genetic study as no clinical suspicion was present at the time of diagnosis.

## Conclusions

The predictive model proposed by the PANOMEN-3 group may be useful to guide the prognosis and therapy of PT in the Spanish population since it offers an acceptable diagnostic accuracy for identifying patients with PT at higher risk of recurrence or biochemical/radiologic progression. Its application in clinical practice could help to tailor follow-up and treatment strategies based on individual risk profiles, ultimately improving patient care and prognosis. Nonetheless, the prognosis of patients with PTs and with grades 2 and 3 in the PANOMEN-3 is similar; thus, these patients should be reclassified in a single common grade.

## Data Availability

Data collected for the study, including individual participant data and a data dictionary defining each field in the set, will be made available to others under reasonable request.

## Abbreviations

ACTH: adrenocorticotropin
ADH: antidiuretic hormone
NFPT: nonfunctioning pituitary tumor
PT: pituitary tumor
TSH: thyrotropin
