# Correction to: “Validation and Limitations of the PANOMEN-3 Predictive Model for Tumor Recurrence and Progression in Pituitary Tumors”

**DOI:** 10.1210/clinem/dgag009

**Published:** 2026-01-22

In the above-named article by Araujo-Castro M, Menéndez Torre E, Lozano-Aida C, García-Centeno R, González Fernández L, Idrobo C, Achote-Rea E, Irigaray Echarri A, Rodríguez-Argulló MDM, Paja M, Guerrero-Pérez F, Castaño JP, Ollero García MD, Novo-Rodríguez C, Tenorio-Jimenéz C, Villar-Taibo R, Bernabeu I, Díaz-López E, Calatayud M, Alvarez-Escola C, Rojas-Marcos PM, Recio-Córdova JM, Aulinas A, Asla Roca Q, Aviles MD, López Mezquita E, Fernández-Argüeso M, González Molero I, Ruz-Caracuel I, Aldecoa I, García-Arabehety J, Martínez-Sáez E, Hanzu F, Marazuela M, Puig-Domingo M, and Biagetti B (*J Clin Endocrinol Metab*. 2025; 110(12): 3391-3399; doi: 10.1210/clinem/dgaf252) there was an error in the author list.

In the originally published article, in the author list, author Julia García Arabehety’s name was incorrectly published as “Julia García.” In the corrected article, the authors’ name has been updated to “Julia García-Arabehety.”

The article has been corrected online.

doi: 10.1210/clinem/dgaf252

